# Computational models for pan-cancer classification based on multi-omics data

**DOI:** 10.3389/fgene.2025.1667325

**Published:** 2025-10-28

**Authors:** Jianlin Wang, Jiao Zhang, Xuebing Dai, Chaokun Yan, Caili Fang

**Affiliations:** School of Computer and Information Engineering, Henan University, Kaifeng, Henan, China

**Keywords:** pan-cancer classification, multi-omics data, deep learning algorithm, convolutional neural network, tumor heterogeneity

## Abstract

Tumor heterogeneity presents a significant challenge in cancer treatment, limiting the ability of clinicians to achieve accurate early-stage diagnoses and develop customized therapeutic strategies. Early diagnosis is crucial for effective intervention, yet current methods lack robust solutions to overcome this challenge. The Pan-Cancer Atlas has emerged as a pivotal framework to investigate cancer heterogeneity by integrating multi-omics data (genomics, transcriptomics, proteomics) across tumor types. This initiative systematically maps inter- and intratumor variations, providing insight for clinical decision making. However, such frameworks often struggle to integrate dynamic temporal changes and spatial heterogeneity within tumors, limiting their real-time clinical applicability. In this review, we first summarize the available multi-omics data and public biomedical databases used in pan-cancer research. Then, we examine current pan-cancer classification approaches based on the computational models they employed, including machine learning and deep learning. We also provide a comparison of these classification methods to explore their advantages and limitations. Finally, we conclude by discussing the key challenges in pan-cancer research and suggesting potential directions for future studies.

## 1 Background

Cancer, a heterogeneous group of diseases that affect various tissues and organs, constitutes a major global health burden. Despite advances in prevention, detection, and therapeutic interventions, global cancer incidence and mortality rates continue to increase (Santucci et al., 2020; Bray et al., 2024). A key limitation of current clinical practices is their reliance on molecularly insensitive tools, which often detect cancer only at intermediate or advanced stages, preventing early diagnosis (Wei et al., 2022). This delay is critical, as early detection is directly related to patient outcomes. For example, the 5-year survival rate for early-stage prostate cancer is 98%, and early breast cancer has a cure rate exceeding 95% (Siegel et al., 2020). However, tumor heterogeneity and similarity complicate early and accurate diagnosis, as well as treatment planning. Tumor heterogeneity manifests itself through genomic, transcriptomic, and proteomic differences between tumor cells, driving variations in morphology, proliferation, and metastatic potential (Zheng et al., 2022). Furthermore, even within the same tumor, cancer cells exhibit phenotypic and morphological heterogeneity during progression (Zhang et al., 2025). For example, lung cancer cells can differentiate into the subtypes of small cell lung cancer, lung squamous cell carcinoma, and lung adenocarcinoma (Yang and Fan, 2024). Each type and subtype of cancer has unique characteristics, leading to various clinical treatment approaches, and this heterogeneity poses significant challenges to diagnosis and treatment (Capper et al., 2018). The similarity of tumors is reflected in the finding that, at a molecular level, tumors in different parts of the body can be more similar than tumors of the same type (Sinha et al., 2021).

To address these challenges, The Cancer Genome Atlas (TCGA) launched the Pan-Cancer Project in 2012 (Weinstein et al., 2013), integrating omics data from more than 11,000 tumor samples to identify shared and unique oncogenic drivers. Pan-cancer aims to describe and identify the commonalities and differences between different types of cancer in order to find the key factors that may trigger cancer and thus guide clinical diagnosis, which is important to improve the cure rate of cancer. Many institutions have launched pan-cancer studies and developed public databases that collect data from various cancer-related researches. For example, the UCSC Genome Browser, that developed and maintained by the University of California, Santa Cruz (UCSC), is a comprehensive multi-omics database. Integrates various types of molecular data including copy number variations, methylation profiles, gene and protein expression levels, and mutation records. Furthermore, the platform supports efficient data analysis and visualization through user-friendly tools. The Gene Expression Omnibus (GEO), developed and maintained by the National Center for Biotechnology Information (NCBI), serves as a public repository for gene expression data. This database systematically integrates diverse cancer-related datasets, including high-throughput gene expression profiles and microarray data. Analysis of these pan-cancer datasets enables researchers to identify unique features of individual cancer types and explore shared or distinct molecular patterns across cancers. Such insights support the accurate classification of cancer subtypes and the development of targeted therapies. These research efforts form the foundation for the advancement of precision cancer and remain a central focus in contemporary cancer studies.

**FIGURE 1:**
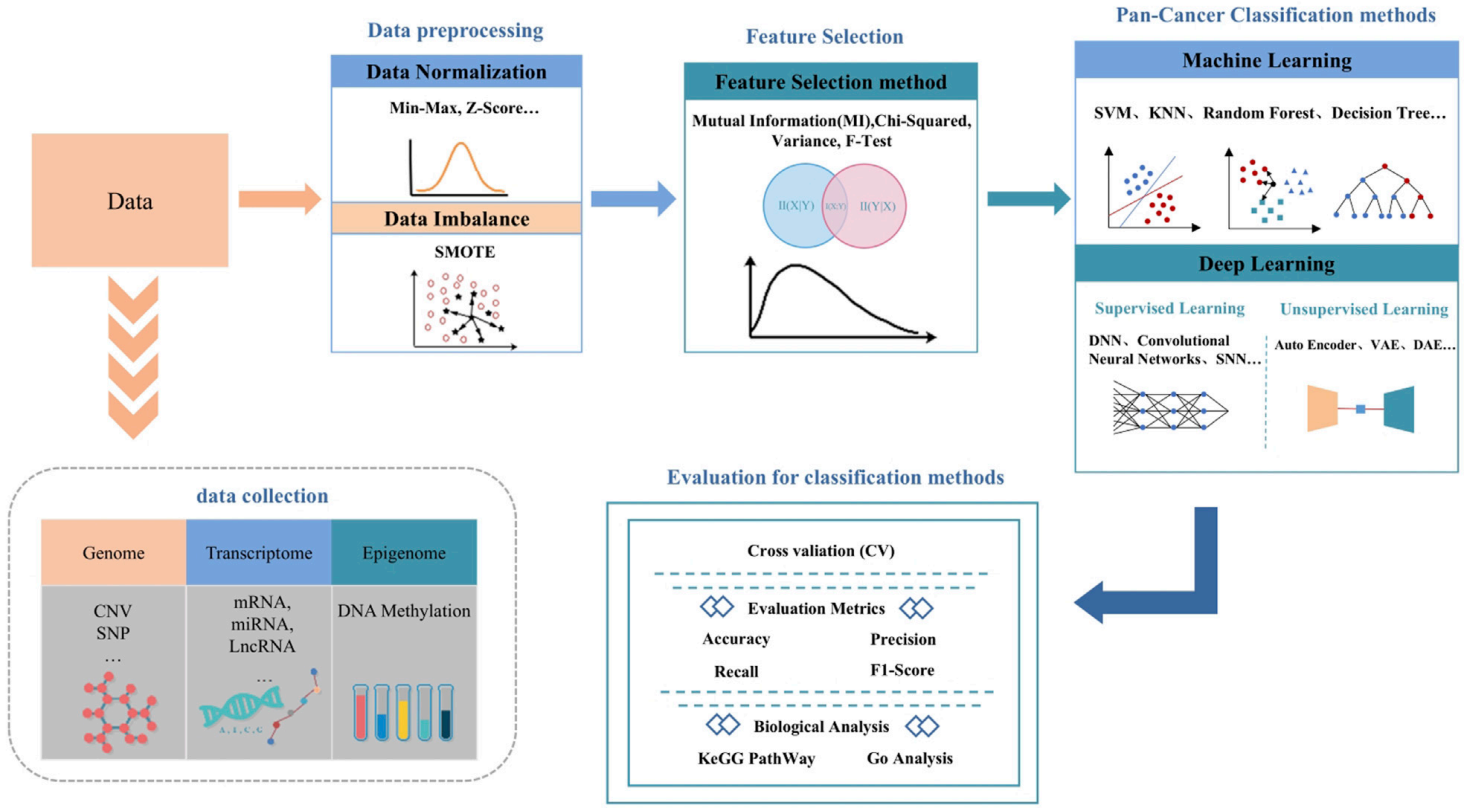
The workflow of pan-cancer classification model.

Traditional pan-cancer studies relied on cluster analysis, network modeling, and pathway enrichment to identify histological similarities. However, these methods lack the resolution required for early diagnosis. Rapid advancements in sequencing technologies have exponentially increased the scale and complexity of omics data, necessitating advanced computational approaches. Machine learning (ML) and deep learning (DL) methods now offer scalable solutions to analyze these high-dimensional datasets. For example, Li et al. (2017) achieved 90% precision in classifying 31 tumor types using genetic algorithms (GA) and K closest neighbors (KNN), while Lyu and Haque (2018) leveraged convolutional neural networks to classify 33 cancers with 95. 59% precision, identification of biomarkers via guided Grad-CAM. Overall, classification studies of pan-cancer datasets are important for improving the cure rate of cancer. Figure 1 shows the standardized workflow for pan-cancer classification models utilizing machine learning and deep learning frameworks.

Initially, researchers must collect and curate data from diverse publicly accessible biomedical databases relevant to the onset and progression of cancer. These data are critical for identifying oncogenic drivers underlying tumorigenesis. With advances in computer technology, a variety of feature dimension reduction and classification algorithms have been developed. These tools are instrumental in constructing models that can accurately discriminate between different cancer types. Once developed, the performance of these methodologies should be assessed against state-of-the-art approaches. This involves comparing them across various metrics and prediction tasks using both standard and supplementary test datasets. Lastly, conducting relevant biological analyses and validations is vital to ensure the reliability and applicability of the findings.

Despite the existence of numerous classification methods for pan-cancer studies, there is a lack of comprehensive literature reviewing the data and methodologies employed. We addresses this gap by providing a thorough analysis of recent pan-cancer classification methods based on diverse models. We begin by exploring the data types commonly used in pancancer research and curating biomedical databases. This process improves our understanding of cancer heterogeneity and similarities and helps to validate research findings. We then examine prevalent classification approaches utilizing machine learning and deep learning models. Finally, we analyze standard datasets and evaluation metrics used in pan-cancer classification and provide a concise comparison of various methods. This comparison aims to assess the strengths and limitations of each approach.

## 2 Data and databases

### 2.1 Available data

With the conclusion of the Human Genome Project and the onset of the post-genomic era, innovative sequencing technologies have emerged (Waterman, 2021). Currently, gene microarray technology and transcriptome sequencing are the primary methods for acquiring cancer multi-omics data. Gene microarray technology, also called DNA microarray, detects both qualitative and quantitative information of DNA or RNA within a sample (Karakach et al., 2010). Transcriptome sequencing (RNA-Seq), also known as second-generation sequencing, offers greater accuracy and sensitivity in gene expression detection compared to microarray technology (Wang et al., 2009). Advancements in sequencing technologies have generated vast multi-omics datasets encompassing genomic, transcriptomic, and proteomic profiles. These multi-omics datasets serve as foundational resources for systematic exploration of oncogenic mechanisms across genomic, transcriptomic, and proteomic dimensions. Subsequently, we provide a detailed description of the multi-omics data closely related to pan-cancer research.

#### 2.1.1 mRNA expression data

mRNA is a single-stranded RNA molecule that carries genetic information transcribed from DNA. It plays a crucial regulatory role in protein synthesis within the cell (Qin et al., 2022). mRNA expression data provide insights into gene function and activity. Investigating fluctuations in gene expression levels can elucidate disease development mechanisms. In cancer research, mRNA expression profiling has emerged as an essential element in elucidating cancer progression mechanisms. Studies show that dysregulation of specific genes can result in uncontrolled cell proliferation, a major factor in cancer development (Leibovitch and Topisirovic, 2018). For example, Li et al. (2017) used GA with a KNN classifier to classify mRNA data from 9,096 tumor samples of 31 types with 90% precision. Similarly, Kim et al. (2020) identified key genes that accurately distinguish 21 types of tumors by using ANOVA tests on mRNA data from cancer and normal samples. Therefore, studying mRNA expression data to find oncogenes helps in early cancer diagnosis and more accurate classification, improving treatment.

#### 2.1.2 miRNA expression data

miRNAs are small noncoding RNAs present in plants and animals, typically 20 to 24 nucleotides long. They play a critical role in the regulation of cellular processes (Cui et al., 2025). miRNA controls oncogenes and tumor suppressor gene expression by degrading mRNAs or inhibiting their translation (Tang et al., 2021; Galagali, 2020). For example, in non-small cell lung cancer, high let-7 expression reduced lung cancer cell growth and inhibited differentiation (Pop-Bica et al., 2020). In gastric cancer, certain miRNAs inhibit the expression of the phosphatase and tensin homolog (PTEN) gene and promote cancer cell growth and invasion (Ashrafizadeh et al., 2020). Wang et al. (2019) combined GA with random forest (RF) for pan-cancer classification of miRNA data from 32 tumor types, achieving 92% sensitivity. To more robust and reliable set of miRNA features capable of distinguishing different types of tumor, Lopez-Rincon et al. (2019). developed an integrated feature selection algorithm for an accfor ante classification of 28 types otypes of tumorsth reliable miRNA features. Therefore, studying miRNA functions is vital for accurate cancer classification and early diagnosis, significantly impacting treatment and prognosis.

#### 2.1.3 lncRNA expression data

lncRNAs are RNA molecules with transcript sequences of more than 200 nucleotides. Although they do not encode proteins, they regulate biological processes such as gene expression, development, and differentiation (Chen et al., 2021). Initially considered as genomic noise, lncRNAs are now recognized as important in cancer development. Changes in their expression can serve as diagnostic markers (Nandwani et al., 2021; Fang and Fullwood, 2016). Analyzing lncRNA data has identified potential biomarkers and distinguished between tumor types (Al Mamun and Mondal, 2019a; Al Mamun and Mondal, 2019b; Al Mamun et al., 2020). Therefore, understanding the roles of lncRNAs is crucial for early cancer diagnosis and treatment.

#### 2.1.4 Copy number variation (CNV)

CNV refers to the variation in the number of copies of a particular gene present in an individual’s genome (Pös et al., 2021). Genes such as BRCA1, CHEK2, ATM, and BRCA2 have strong associations with cancers like breast cancer (Hu et al., 2018). Zhang et al. (2016) proposed using a Dagging classifier to categorize CNV data from six cancer types, highlighting key features for accurate classification. Therefore, studying CNV helps explore cancer pathogenesis, aiding early diagnosis and treatment selection.

#### 2.1.5 DNA methylation

DNA methylation, an epigenetic modification, involves adding a methyl group to DNA, usually suppressing gene expression (Liu et al., 2016). It is crucial for normal cellular functions and implicated in cell differentiation and tumorigenesis. Dysregulated methylation, such as hypermethylation of CpG islands in promoter regions, can silence tumor suppressor genes or reduce oncogenic miRNA transcription, increasing cancer risk (Formosa et al., 2013). Liu et al. (Liu et al., 2019) used methylation data from 27 cancers types and proposed machine learning and deep learning strategies for accurate cancer differentiation. Therefore, DNA methylation is closely related to the occurrence and development of cancer, and the analysis and study of methylation is very important in the field of cancer diagnosis.

#### 2.1.6 Multi-omics

The development of cancer is a very complex process that is not simply caused by the occurrence of abnormalities in one type of data, but often involves multiple histological pathological processes. Therefore, data mining analysis based on single omic data has certain one-sidedness and limitations. In recent years, with the rapid development of next-generation genomic technologies, a large amount of genomic data of different types of cancers has been accumulated, and more and more researchers have started to integrate multiple omic data to conduct systematic and complete analysis of the mechanisms of cancer occurrence, and cancer research is developing from single omic to multi-omics. Integrated multi-omics analysis can make up for the lack of information in single-omics data and provide a comprehensive view of patients, and enable researchers to explore the relationship between cancer and genes from multiple perspectives, so as to perform early cancer diagnosis more accurately.

Table 1 summarizes the characteristics of common pan-cancer data types, including mRNA, miRNA, and DNA methylation.

**TABLE 1:** Description of common data types of pan-cancer. The dimensions presented are the feature counts derived from the TCGA Pan-Cancer Atlas dataset.

Data type	Data description	Dimension
mRNA	The real-time product of gene expression, which controls protein synthesis, and abnormal expression can lead to the development of cancer	20,531
miRNA	Key molecules in the regulation of transcription and translation of oncogenes and tumor suppressor genes. Aberrant expression regulates tumor cell growth, proliferation and apoptosis	1,882
lncRNA	An RNA molecule that does not have protein-coding ability and is involved in the development of cancer, and changes in its expression level can be used as a marker for the diagnosis of cancer	19,166
CNV	Caused by genomic rearrangements, occurring in genes 1-kb or longer in length that are implicated in the development and progression of human cancers	24,174
DNA Methylation	DNA methylation usually inhibits the expression of genes in cells and plays an important regulatory role, and abnormal expression silences tumor suppressor genes leading to the development of cancer	48,578

### 2.2 Biomedical database

With the rapid development of high-throughput sequencing technology, a large amount of tumor-related histological data has been accumulated, and meanwhile, various public medical databases have emerged continuously. These public databases can be classified into comprehensive databases, genomic, transcriptomic, epigenomic databases, etc. according to the research areas or data types. Table 2 summarizes some cancer-related databases and provides brief descriptions and access links.

**TABLE 2:** Overview of the cancer database.

Database	Brief description	Links
TCGA (Weinstein et al., 2013)	Collected multiple omics data of 33 tumor types, the largest human tumor sequencing database in the world	https://www.cancergenome.nih.gov/
EGA (Lappalainen et al., 2015)	Collection of over 800 medical studies of all types of sequencing data and typing data	https://ega-archive.org/
CGHub (Wilks et al., 2014)	Sequencing data of 25 different types of cancers from TCGA, TARGET, and CCLE were collected and organized	https://cghub.ucsc.edu/
ICGC (Consortium et al., 2010)	Collecting omics data from many different types of cancers, and comprehensively described the genomic changes of many cancer	https://dcc.icgc.org/
COSMIC (Forbes et al., 2015)	Collecting omics data on many types of cancer, it is the world’s largest and most comprehensive database of somatic mutations	https://cancer.sanger.ac.uk/cosmic
cBioPortal (Gao et al., 2013)	Collects genomic data on many different types of cancer, providing visual analysis tools across genes, samples and data types	http://www.cbioportal.org/
UCSC Xena (Navarro Gonzalez et al., 2021)	Collecting data from several large cancer research projects, and provides convenient data analysis and visualization capabilities	http://genome.ucsc.edu/
arrayMap (Cai et al., 2015)	Provide pre-processed tumor genome microarray data and CNA atlas	http://www.arraymap.org/
BioMuta (Wu et al., 2014)	26 different types of cancers were collected SNV-data	https://hive.biochemistry.gwu.edu/home/
GEO (Barrett et al., 2012)	Collection and organization of high-throughput gene expression data submitted by research institutions around the world	https://www.ncbi.nlm.nih.gov/geo/
ArrayExpress (Kolesnikov et al., 2015)	Collected and organized microarray chip-based and high-throughput sequencing of experimental genomics data	https://www.ebi.ac.uk/arrayexpress/
OncomiRDB (Wang et al., 2014)	Collection and annotation of experimentally validated miRNAs with promotive or inhibitory effects on different cancer types	http://www.oncomir.org/
miRCancer (Xie et al., 2013)	A comprehensive collection of miRNA expression profiles in various human cancers	http://mircancer.ecu.edu/
SomaMiR (Bhattacharya et al., 2013)	Collecting data on miRNAs and mutations on their targets	https://compbio.uthsc.edu/SomaMiR/
ChiTaRS (Frenkel-Morgenstern et al., 2015)	Cancer genome sequence breakpoints were collected along with expression level data of the corresponding chimeric transcripts	https://chitars.bioinfo.cnio.es/
MethylCancer (He et al., 2007)	Collected tumor DNA methylation, cancer-related genes, mutations, CpG islands, and cancer information	http://methylcancer.psych.ac.cn/
MethHC (Huang et al., 2015)	Organized DNA methylation, mRNA/miRNA gene expression, miRNA methylation, and association between methylation and gene expression levels from TCGA	http://methhc.mbc.nctu.edu.tw/
CGC (Subramanian et al., 2021)	NCI-funded cloud platform co-localizing large datasets, and compute power for secure, collaborative multi-omics analysis	https://www.cancergenomicscloud.org/
CPTAC (Mesri et al., 2024)	CPTAC provides a rich source of public data, serving as a critical resource for researchers studying pan-cancer proteomics	https://cptac-data-portal.georgetown.edu/cptac/

Next, we provide a detailed description of the most commonly used databases in pan-cancer research.

#### 2.2.1 TCGA

TCGA is the largest human tumor genome sequencing database globally (Weinstein et al., 2013). Jointly sponsored by the National Human Genome Research Institute (NHGRI) and the National Cancer Institute (NCI), this major research project was officially launched in 2005. TCGA has sequenced 33 common cancers and over 11,000 tumor samples, using genomic analysis technology to enhance understanding of tumor mechanisms and improve cancer diagnosis and treatment capabilities (Tomczak et al., 2015). TCGA currently provides mRNA expression data, miRNA expression data, DNA methylation data, CNV data, and other high-throughput sequencing data. Researchers can access these datasets through the Genomic Data Commons (GDC) Data Portal, the primary data source for many cancer researchers.

#### 2.2.2 GEO

GEO is a subdatabase of the National Center for Biotechnology Information (NCBI). This free and publicly accessible repository houses biological data from gene chips, second-generation sequencing, and other high-throughput functional genomics experiments. It includes submissions from over 16,000 laboratories and research teams worldwide, featuring 175,825 datasets with 5,069,606 data samples. GEO supports data download capabilities, enabling users to obtain samples or datasets of interest. Additionally, it offers tools to discover genes of interest and their expression profiles, as well as to identify genes with similar expression patterns.

#### 2.2.3 UCSC Xena

UCSC Xena is a cancer genomics data analysis platform developed by the UCSC Cancer Genome Browser (Navarro Gonzalez et al., 2021). This platform collects and standardizes data from several major cancer research projects such as TCGA, ICGC, and TARGET, facilitating subsequent analysis (Consortium et al., 2010). UCSC Xena encompasses multiple levels of data, including copy number, methylation, gene expression, protein expression, and mutation data. It provides user-friendly data analysis and visualization tools. Researchers can easily analyze or download organized data with link clicks and can also upload their data for analysis. This flexibility considerably aids in the advancement of genomic research.

#### 2.2.4 CPTAC

The Clinical Proteomic Tumor Analysis Consortium (CPTAC) is a comprehensive proteomic and genomic research program initiated by the National Cancer Institute (NCI) that aims to accelerate the understanding of cancer biology through the integration of large-scale proteomic and genomic analysis (Mesri et al., 2024). The consortium systematically identifies, quantifies, and analyzes proteins from cancer biospecimens characterized by genomic data to improve cancer prevention, early diagnosis, treatment, and prognosis. CPTAC provides a rich source of public data, serving as a critical resource for researchers studying pan-cancer proteomics. Its data, which includes protein abundance, post-translational modifications, and mass spectrometry data, is often used in combination with genomic data to provide a multi-layered view of tumors, enabling the discovery of new biomarkers and therapeutic targets.

#### 2.2.5 CGC

The Cancer Genomics Cloud (CGC), an NCI-funded resource powered by Seven Bridges, is a secure and scalable cloud-based platform designed to overcome the challenges associated with accessing, sharing, and analyzing massive, diverse multi-omics datasets (Subramanian et al., 2021). The platform achieves this by co-localizing three essential components within the cloud: major cancer datasets like The Cancer Genome Atlas (TCGA) and Clinical Proteomic Tumor Analysis Consortium (CPTAC); over 400 bioinformatics tools and best-practice workflows; and the high-performance computational capabilities for large-scale analysis. The CGC simplifies the user experience by enabling researchers to browse, query, and filter datasets, run their entire analysis workflow on the platform, and even integrate their own private tools and data.

Building on the data sources described above, the following section reviews computational methods for pan-cancer classification.

## 3 Methods

**FIGURE 2:**
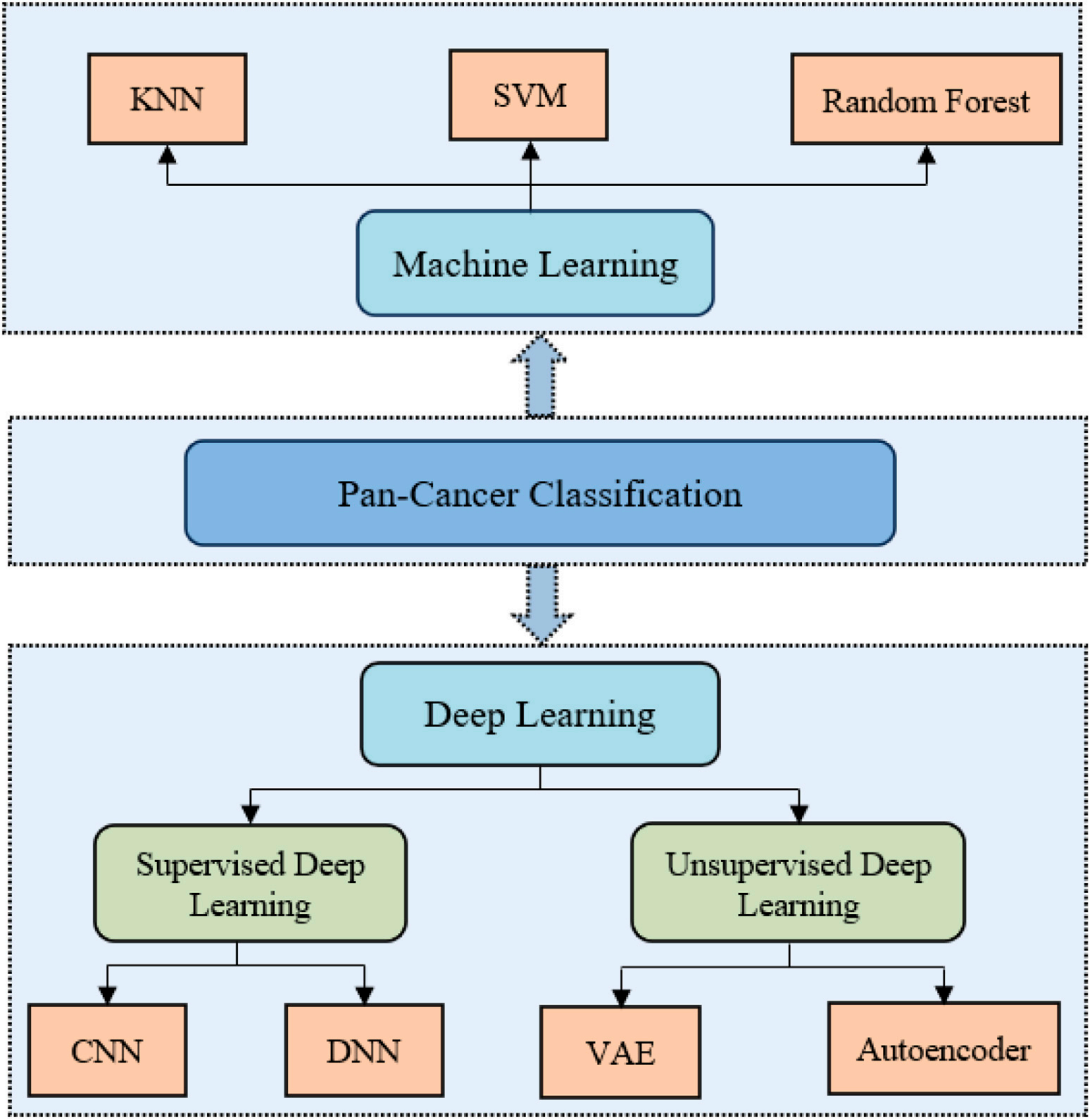
Pan-Cancer classification methods based on various models.

Advances in biotechnology have significantly expanded the application of gene sequencing in pan-cancer studies. The proliferation of high-throughput sequencing data offers a critical foundation for research. However, a key challenge lies in developing efficient computational algorithms to extract biologically meaningful insights from these complex datasets. Current methodologies for pan-cancer analysis are broadly categorized into two frameworks: classical machine learning and deep learning. As illustrated in Figure 2 deep learning models can be further subdivided into supervised and unsupervised approaches, depending on the utilization of labeled data.

### 3.1 Pan-cancer classification model based on machine learning

Feature selection innovations and model optimization strategies in machine learning have significantly advanced pan-cancer classification accuracy. To balance feature relevance and parsimony, Kim et al. (2020) implemented a two-stage gene selection strategy: ANOVA-based F-statistic ranking identified top genes across 21 cancers, followed by frequency-based filtering. Neural networks trained on 300 selected genes achieved peak accuracy (90%), outperforming other classifiers. Mahin et al. (2022) refined this approach by retaining only genes consistently expressed across all 21 cancers and incorporating data smoothing/oversampling, enhancing model robustness. Luo et al. (2023) developed an ML approach to predict cancer prognosis considering 32 cancer types from TCGA.Initially, the approach was applied to hepatocellular carcinoma and then extended to other types of tumors.

Beyond conventional methods, researchers have explored hybrid and multi-algorithm frameworks. Khadirnaikar et al. (2023) analyzed mRNA, miRNA, DNA methylation, and protein of 33 different types of cancer from TCGA. Firstly, multi-omics data was combined by concatenating the features for each sample, and then the autoencoder was used to reduce the dimension of data. Novel subtypes of cancer samples were identified by clustering k-means. Further exploring the efficacy of the classifier, Elsadek et al. (2019) employed a machine learning approach using gene CNV data across six types of tumor. Their approach utilized an information gain algorithm for gene selection and evaluated various classifiers, with LR achieving superior performance, underscoring machine learning’s role in cancer classification. Liu (2022) analyzed the association with a correlation test of epi-driver CpG sites between DNA methylation and gene expression profiles. XGBoost and SHAP algorithms identified the best biomarkers in five genes and used them as features for the generation of a random forest model to identify cancer subtypes. Finally, Cheerla and Gevaert (2017) and Al Mamun and Mondal (2019a) both explored two-stage feature selection approaches. Cheerla’s team reduced miRNA features using correlation and recursive elimination, achieving the best classification with SVM radial among 21 tumor types. Mamun’s approach selected common features for classifiers, finding SVM provided the best accuracy for eight different cancers. Collectively, these innovations underscore machine learning’s adaptability in addressing omics complexity while balancing feature parsimony and accuracy.

### 3.2 Pan-cancer classification model based on deep learning

Although machine learning methods have been widely used to study pan-cancer classification problems and achieved good results, with the development of deep learning and the high performance shown on classification tasks, more and more researchers have started to use deep learning to improve the performance of tumor classification models. In the field of deep learning, deep learning methods can be classified into two categories based on whether the models use the labels of the data, namely, supervised learning and unsupervised learning (Alzubaidi et al., 2021).

#### 3.2.1 Supervised classification models

Recent advancements in supervised deep learning have demonstrated remarkable efficacy in pan-cancer classification through tailored architectural innovations. Sun et al., 2018) introduced GeneCT, an artificial neural network (ANN) framework designed to classify 11 tumor types using raw mRNA expression data without feature engineering, achieving 98.2% accuracy and underscoring the potential of end-to-end learning in omics analysis. Complementing this approach (Cava et al., 2023), applied principal component analysis (PCA) to reduce data dimensionality before deploying the model. The neural network achieved a mean accuracy of 84%, the random forest reached 86%, and XGBoost achieved the highest performance with a mean accuracy of 90%. To address the challenges of limited sample sizes in specific cancer types (Cho et al., 2023), proposed a meta-learning method that integrates multi-omics data (transcriptomics, proteomics, and clinical data from TCGA) to create predictive models using survival information from 17 cancer types. Their approach requires fewer samples than conventional deep learning models, effectively mitigating data scarcity issues. Expanding this paradigm (Divate et al., 2022), employed deep neural networks (DNNs) to classify 33 cancer types. Their methodology integrated expression-based gene screening with SHAP (Shapley Additive exPlanations) interpretability, identifying critical biomarkers and achieving superior performance in distinguishing cancers from healthy controls.

To address high-dimensional data challenges (Wu et al., 2024) developed DeepMoIC, a method combining deep graph convolutional networks (GCNs) with autoencoders for cancer subtype classification. By constructing a patient similarity network (PSN) and leveraging GCNs, DeepMoIC outperformed existing models on multi-omics datasets, highlighting its potential for precision oncology. (Li et al., 2025) introduced DGHNN, a deep graph and hypergraph neural network for pan-cancer related gene prediction that takes biological pathways into consideration. This method applies a deep graph and hypergraph neural network to encode higher-order information in protein interaction networks and biological pathways. This approach, along with the introduction of skip residual connections and a feature tokenizer with a transformer for classification, demonstrates how advanced network architectures can capture the multi-level complexity of biological systems, setting a new standard for performance. (Li et al., 2020) tackled CNV sparsity by coupling Monte Carlo feature selection (MCFS), which evaluates feature stability via randomized sampling, with self-normalizing neural networks (SNNs) to enhance training robustness. Their framework achieved 79.8% accuracy in classifying four cancer types. These studies collectively highlight how supervised architectures can be customized to diverse omics modalities while balancing performance and biological interpretability.

In recent years, due to the excellent performance of convolutional neural networks (CNNs) on image classification tasks, more and more researchers have started to apply these networks to the classification problem of pan-cancer. For instance (Ameen et al., 2025) proposed a stacked deep learning ensemble model for multi-omics cancer type classification, demonstrating that deep learning can be effectively applied to high-dimensional biological data. Similarly (Lyu and Haque, 2018), firstly proposed the use of a convolutional neural network to classify mRNA expression data by embedding high-dimensional gene expression data into a two-dimensional image as the input of the convolutional neural network to classify 33 different types of tumors. Building on this, Mostavi et al. (Mostavi et al., 2020) systematically compared CNN architectures (e.g., Inception modules, residual connections), revealing that deeper networks achieved 95. 82% precision on 33-class tasks that highlight the impact of structural optimization. Addressing computational inefficiency Khalifa et al., 2020), applied binary particle swarm optimization (BPSO) to reduce the dimensionality of mRNA from 20,531 to 512 features before CNN training, achieving accuracy of 96. 9% on five types of tumors. Hybrid models also emerged as a promising frontier: (Huynh et al., 2019) combined deep CNNs (DCNN) with SVM classifiers, where DCNNs extracted high-order features and SVMs performed classification, reaching 76. 33% precision for 25 cancers. (Abdullahi et al., 2020) further demonstrated the efficiency of fine-tuning pre-trained AlexNet models on mRNA data, reaching 98.1% accuracy for five cancers with minimal computational overhead. Beyond expression data (Ye et al., 2021) encoded somatic mutation profiles into heatmap-like “mutation maps,” enabling ResNet-50 and Inception-v3 models to outperform traditional methods (89.7% vs. SVM’s 72.3%). Finally (AlShibli and Mathkour, 2019) validated CNNs’ versatility in CNV analysis, showing that a six-layer residual network (ResCNN6) surpassed standard CNNs and VGG-16 (86% accuracy for six cancers), underscoring the efficacy of residual connections in combating gradient vanishing. These innovations exemplify CNNs’ adaptability to multi-omics integration through data transformation, architectural refinement, and cross-domain transfer learning.

#### 3.2.2 Unsupervised classification models

Unsupervised deep learning techniques have emerged as powerful tools for pan-cancer classification, particularly in scenarios with limited labeled data. Rong et al. (Rong et al., 2022) proposed a computational approach, multi-omics clustering variational autoencoders (Mcluster-VAEs), based on a new probabilistic model of a deep learning method consisting of clustering algorithm for multi-omics data to estimate posterior cancer subtypes. Building on this (Al Mamun et al., 2020) introduced the Concrete Autoencoder (CAE), an unsupervised framework for identifying discriminative lncRNAs. The CAE outperformed supervised methods (Lasso, RF, SVM-RFE) in classifying 33 tumors, achieving 93% accuracy. To address feature instability across CAE iterations (Al Mamun et al., 2021) later proposed the multi-run CAE (mrCAE), which aggregated high-frequency lncRNAs from 100 CAE runs to derive a stable subset of 69 markers. This refined set enabled accurate classification of 12 cancers, resolving reproducibility challenges inherent to stochastic deep learning models. Expanding to multi-omics integration (Zhang et al., 2019) developed OmiVAE, an end-to-end model combining VAEs with a classification network. OmiVAE first compressed the mRNA and DNA methylation data into low-dimensional embeddings, then predicted 33 tumor types using a three-layer neural network, achieving precision of 97. 49%. Finally (Albaradei et al., 2021) designed MetaCancer, which used convolutional VAE to extract features from mRNA, miRNA and methylation data. When fed into a deep neural network (DNN), this multi-omics integration classified 11 cancers with 88.85% accuracy-surpassing mRNA-only approaches by 14.2%. (Li et al., 2024) proposed AVBAE-MODFR, a two-phase framework that combines adversarial variational Bayes autoencoder for multi-omics embedding with a dual-net feature ranking module. Tested on TCGA pan-cancer data, AVBAE-MODFR outperformed four state-of-the-art methods, highlighting its robustness in representation learning and biomarker discovery. Compared with earlier VAE-based models such as OmiVAE and MetaCancer, AVBAE-MODFR not only integrates heterogeneous omics but also incorporates an explicit feature ranking mechanism, thereby enhancing interpretability and facilitating the identification of biologically meaningful markers. These innovations underscore unsupervised learning’s potential to uncover robust biomarkers and integrate heterogeneous omics data without reliance on labeled datasets.

**FIGURE 3:**
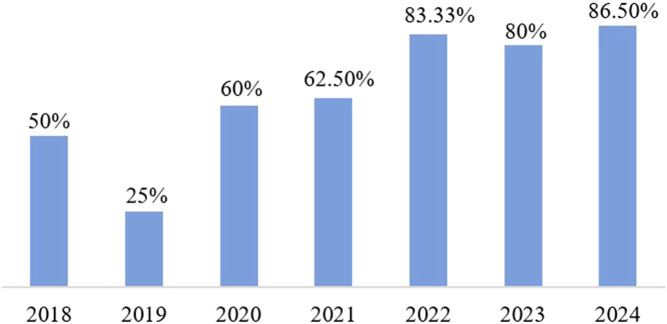
The ratio of pan-cancer research using deep learning technologies.A systematic review of the relevant literature shows a steady increase in the use of deep learning in pan-cancer research in recent years.

Figure 3 illustrates the growing prominence of deep learning in pan-cancer research. It shows the percentage of all pan-cancer-related articles that used deep learning methods for classification over the past few years. A systematic review of papers published on the PubMed and Web of Science platforms using search terms “pan-cancer classification”, “deep learning” and “machine learning” from 2018-2024 revealed a steady increase in this ratio from 2018 to 2024. To summarize the current landscape of pan-cancer classification, we present an overview of relevant studies in recent years in Table 3. This table highlights the variety of machine learning and deep learning approaches, as well as the multi-omics data they employ.

**TABLE 3:** Overview of pan-cancer classification methods.

References	Method type(s)	Data type(s)	Data source	Cancer types	Code link
Kim et al. (2020)	ML (SVM,KNN)	mRNA	–	21	–
Mahin et al. (2022)	ML (KNN)	mRNA	TCGA	22	https://github.com/Zwei-inc/panclassif
Luo et al. (2023)	ML (SVM)	Gene expression	TCGA	32	–
Khadirmaikar et al. (2023)	ML (SVM)	mRNA, miRNA,DNA Methylation	GDC	33	https://github.com/seemark11/Pancancer-subgroup-identification
Cheerla and Gevaert (2017)	ML (SVM)	miRNA	–	21	–
Al Mamun and Mondal, (2019a)	ML	lncRNA	–	8	–
Elsadek et al. (2019)	ML (SVM,Random forest)	CNV	–	6	–
Liu (2022)	ML (Random forest)	DNA Methylation, Gene expression	–	11	–
Sun et al. (2018)	SDL	mRNA	–	11	http://sunlab.cpy.cuhk.edu.hk/GeneCT/
Cava et al. (2023)	SDL (Neural Network)	Gene expression	–	16	https://github.com/claudiacava/Applied-Sciences
Cho et al. (2023)	SDL (Neural Network)	Gene expression	TCGA	17	https://github.com/berkuva/TCGA-omics-integration
Mostavi et al. (2020)	SDL (CNN)	mRNA	–	33	https://github.com/chenlabgccri/CancerTypePrediction
Khalifa et al. (2020)	SDL (CNN)	mRNA	–	5	–
Huynh et al. (2019)	SDL (DCNN)	mRNA	–	25	–
Abdullahi et al. (2020)	SDL (CNN)	mRNA	–	5	–
Li et al. (2020)	SDL (SNN)	CNV	–	4	https://github.com/KohTseh/CancerClassification
Rong et al. (2022)	DL	miRNA,DNA methylation,CNV	UCSC	32	https://github.com/luyiyun/MCluster-VAEs
Albaradei et al. (2021)	UDL (CVAE)	mRNA, miRNA,DNA Methylation	–	11	https://github.com/SomayahAlbaradei/MetaCancer
Al Mamun et al. (2020)	UDL (CAE)	lncRNA	–	33	–
Al Mamun et al. (2021)	UDL (CAE)	lncRNA	–	12	–
Zhang et al. (2019)	UDL (VAE)	mRNA,DNA Methylation	UCSC	33	https://github.com/zhangxiaoyu11/OmiVAE
Li et al. (2024)	UDL (VAE/CVAE)	mRNA, miRNA,DNA Methylation	–	33	https://github.com/zhanglabNKU/AVBAE-MODFR

ML: machine learning; SDL: supervised deep learning; UDL: unsupervised deep learning; CNN: convolutional neural network; SNN: self-normalizing neural network; CVAE: convolutional variational autoencoder; CAE: concrete autoencoder; VAE: variational autoencoder.

### 3.3 Integration strategies

The integration of multi-omics data is a critical step in pan-cancer research, as it provides a more comprehensive view of cancer’s molecular mechanisms by combining information from multiple platforms. Integration strategies are typically categorized by the stage at which the data is combined. For instance, an early integration approach, where mRNA and CNV data are simply concatenated, may be easy to implement but can lead to a high-dimensional feature space and potentially introduce noise (Zhao et al., 2024). In contrast, an intermediate integration approach using a variational autoencoder (VAE) to create a joint latent space can handle the high dimensionality and may reveal more complex, underlying relationships between omics types, but the learned features are often less interpretable.

To better evaluate the performance of these pan-cancer classification models, researchers are developing new benchmarks. These include integrating multi-omics data from large consortia, assessing cross-cohort generalization, and shifting the focus to more specific clinical endpoints beyond simple cancer type classification. For example, integrating genomics from TCGA with proteomics from CPTAC offers a more comprehensive understanding of cancer’s molecular mechanisms, as proteins are the functional molecules that execute cellular processes. A related large-scale multi-omics benchmark, CMOB, integrates data from the TCGA platform, providing an accessible and usable resource for machine learning research (Yang et al., 2024).

Beyond these comprehensive datasets, evaluating a model’s generalization ability across different patient cohorts is essential for validating its robustness and reliability in diverse clinical settings. In addition, new benchmarks are moving beyond the simple classification of cancer types to include more refined clinical endpoints such as subtype classification, stage prediction, survival analysis, and prediction of response to treatment. These more granular predictions are crucial for personalized medicine, as they inform specific patient care strategies. Several recent case studies highlight these advances. AVBAE-MODFR is a deep learning framework that integrates multi-omics data through embedding and feature selection, showing potential clinical applications in tumor diagnosis and precision medicine (Li et al., 2024). TMO-Net is another model that is pre-trained on multi-omics pan-cancer datasets to facilitate cross-omics interactions and enable joint representation learning and inference on incomplete omics data, thereby supporting various downstream oncology tasks (Wang et al., 2024).

Future research is also expanding to incorporate new data types and modalities that offer a more holistic view of tumor biology. Single-cell multi-omics (e.g., scRNA-seq, scATAC-seq) provides an unprecedented resolution of tumor heterogeneity at the cellular level, capturing differences between individual cells that are lost in bulk omics data. In addition, integration of radiology and pathology images with molecular data is a promising area. This represents a different data modality (unstructured images) that requires specialized models such as CNNs. Combining these visual cues with molecular data can provide a more comprehensive view of the tumor, bridging the gap between molecular mechanisms and the morphological features observed in clinical practice.

## 4 Evaluation and discussion

### 4.1 Selection criteria

We systematically reviewed papers published on the Ovid and Web of Science platforms. Our search criteria focused on machine learning and multi-omics data for pan-cancer studies. We only included full-text, English-language papers from peer-reviewed journals that used artificial intelligence to analyze multi-omics data on cancer samples. We excluded any papers that only applied machine learning to a single cancer type or data type, did not use cancer samples, or were themselves reviews or proceedings.

### 4.2 Classification evaluation metrics

Classification performance evaluation metrics are essential to objectively assess the effectiveness of classification models. Selecting a high-performing classifier relies on using rigorous evaluation criteria. Accuracy is a common metric for evaluating overall model performance in classification tasks. However, in pan-cancer classification, sample size imbalance is a prevalent issue, where some cancer types have many samples while others have few. In such cases, the majority class can disproportionately influence overall accuracy, diminishing its evaluative significance. For example, a model trained on an imbalanced dataset might achieve a deceptively high accuracy simply by correctly classifying all samples from the majority class, while failing to identify samples from the rarer, minority classes. Thus, relying solely on accuracy is insufficient.

Therefore, it is necessary to consider other metrics that provide a more complete picture of a model’s performance on multi-label, imbalanced datasets. We analyze several evaluation metrics as reported in the reviewed literature, including Precision (PR), Recall (RC), F1-score, Area Under the Receiver Operating Characteristic Curve (AUC), and Matthews Correlation Coefficient (MCC). Precision measures the proportion of true positive predictions among all positive predictions, while recall measures the proportion of true positives correctly identified from all actual positives. The F1-score provides a single value that balances both precision and recall, making it particularly useful for evaluating models on imbalanced data. The AUC and MCC are also important for assessing overall performance, with MCC providing a balanced measure that accounts for all four values in a confusion matrix, regardless of class size.

### 4.3 Data sets

For pan-cancer classification research, multiple of the following 33 cancer types are commonly used for analysis. The types and sample information of these 33 cancers are shown in Table 4.

**TABLE 4:** Types of cancer and number of samples.

No.	Cancer name	Code	Cases
1	Adeno-cortical carcinoma	ACC	79
2	Bladder-Urothelial-Carcinoma	BLCA	408
3	Breast-invasive carcinoma	BRCA	1093
4	Cervical and endocervical cancers	CESC	304
5	Cholangiocarcinoma	CHOL	36
6	Colon-adenocarcinoma	COAD	457
7	Lymphoid-Neoplasm-Diffuse-Large B-cell-Lymphoma	DLBCL	48
8	Esophageal carcinoma	ESCA	184
9	Glioblastoma multiforme	GBM	160
10	Head and Neck squamous cell carcinoma	HNSC	520
11	Kidney-Chromophobe	KICH	66
12	Kidney renal clear cell carcinoma	KIRC	533
13	Kidney renal papillary cell carcinoma	KIRP	290
14	Acute-Myeloid Leukemia	LAML	179
15	Brain Lower-Grade Glioma	LGG	516
16	Liver-hepatocellular carcinoma	LIHC	371
17	Lung adenocarcinoma	LUAD	515
18	Lung squamous cell carcinoma	LUSC	501
19	Mesothelioma	MESO	87
20	Ovarian serous cystadenocarcinoma	OV	304
21	Pancreatic adenocarcinoma	PAAD	178
22	Pheochromocytoma and Paraganglioma	PCPG	179
23	Prostate-adenocarcinoma	PRAD	497
24	Rectum-adenocarcinoma	READ	166
25	Sarcoma	SARC	259
26	Skin Cutaneous Melanoma	SKCM	469
27	Stomach adenocarcinoma	STAD	415
28	Testicular Germ Cell Tumors	TGCT	150
29	Thyroid carcinoma	THCA	501
30	Thymoma	THYM	120
31	Uterine Corpus Endometrial Carcinoma	UCEC	545
32	Uterine Carcinosarcoma	UCS	57
33	Uveal Melanoma	UVM	80

**FIGURE 4:**
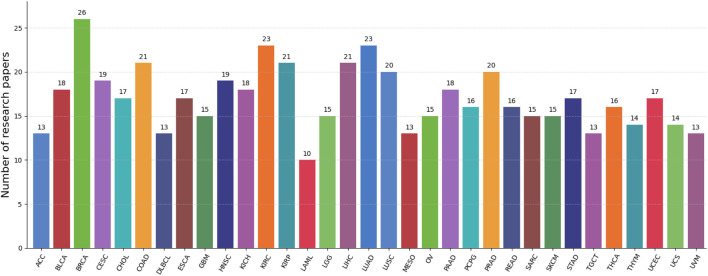
Frequency of cancer types used in pan-cancer classification studies reviewed in this paper. The x-axis indicates the specific cancer types, while the y-axis shows the number of research papers that utilized each cancer type’s dataset. The data presented here is based on a statistical analysis of the literature reviewed in this manuscript.

Next, the analysis performed in terms of datasets employed by the distinct research works is elaborated. Figure 4 depicts several datasets utilized for pan-cancer classification. BRCA is the most frequently utilized dataset in pan-cancer classification research. In addition, the most commonly used datasets in pan-cancer classification also include KIRC, LUAD, COAD, KIRP, LIHC, etc.

### 4.4 Comparison and analysis

As reported in the reviewed literature, a performance comparison of various pan-cancer classification methods on the mRNA gene expression dataset for 33 cancer types reveals that deep learning models generally achieve higher classification accuracies than traditional machine learning methods. For instance (Lyu and Haque, 2018) reported a 95.59% accuracy using a convolutional neural network, a performance that surpasses many of the reported accuracies of traditional machine learning algorithms on similar tasks. This qualitative comparison of architectures suggests that deep learning models are often more capable of distinguishing between 33 different cancer types due to their ability to learn complex, hierarchical features from high-dimensional data.

**FIGURE 5:**
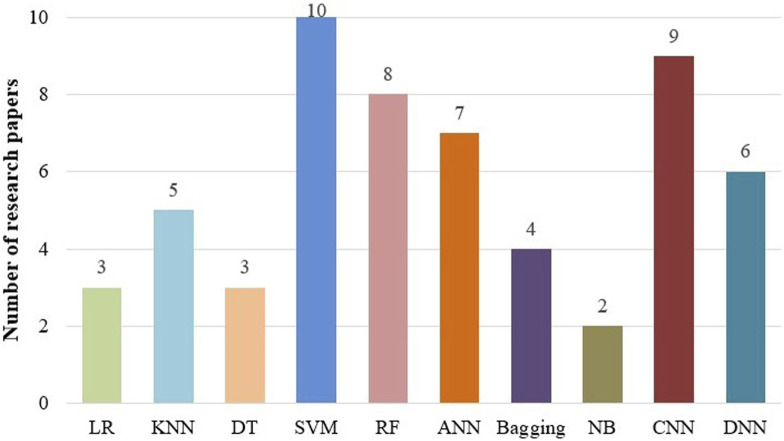
The frequency of different classifiers used in the pan-cancer classification research reviewed in this paper. Here, DNN refers to fully connected architectures (e.g., multilayer perceptrons), excluding convolutional neural networks (CNNs).

Next, the classifiers used in different research works are elaborated and analyzed. Figure 5 illustrates several common classifiers utilized for pan-cancer classification. This figure was generated by counting the primary classifiers used in the reviewed articles. A classifier was counted if it was the main model for the classification task. The raw counts were then converted to percentages to show the proportion of each classifier type. As shown in the figure, the most frequently used machine learning classifiers in pan-cancer classification studies are SVM, RF, ANN, and KNN, respectively. Meanwhile, among deep learning classifiers, CNNs and fully connected deep neural networks (DNNs, e.g., multilayer perceptrons) were the most frequently used.

### 4.5 Discussion

In our review, we have summarized the diverse ML and DL algorithms applied to pan-cancer multi-omics analysis. In many cases, proposed methods were evaluated against existing algorithms, often showing comparable levels of performance. However, no systematic comparison of different approaches on a common dataset has yet been conducted. Despite the variety of methods, there is still no standardized framework applicable in clinical practice. A major challenge remains the difficulty of generalizing results across studies and ensuring reproducibility. To address this, automatic and standardized methodologies that can be readily applied by non-expert users should be developed to better support clinical decision-making.

The application of ML and DL to multi-omics data also presents significant challenges. As multi-omics data derived from different platforms have varying distributions, this must be carefully considered before data integration (Reel et al., 2021). Furthermore, the integration of multiple omics datasets can generate noise and introduce redundant information. New algorithms must also be designed to effectively handle missing observations, as samples may be absent in one or more omics datasets (Leng et al., 2022).

In addition, class imbalance and overfitting are commonly reported issues in biomedical datasets. A training set composed of imbalanced classes can negatively influence the accuracy of a classifier, necessitating the use of statistical techniques such as under- or oversampling (Misra et al., 2019). Moreover, the high-dimensional nature of multi-omics features can impact a classifier’s performance, as correlated features introduce redundant information. To address this, optimal feature selection algorithms should be applied to select a limited, yet representative, subset of features.

## 5 Challenges and future work

Current pan-cancer classification methods leverage diverse data types and models to improve cancer type differentiation and inform clinical decision-making. This review systematically summarizes the methodologies, data sets, and evaluation metrics used in pan-cancer research, highlighting the progress in utilizing genomics, transcriptomics, and epigenomics to analyze tumor heterogeneity. We reviewed current pan-cancer classification methods, categorizing them based on the models used and assessing their performance across different data types.

Despite these advancements, challenges persist. Many models heavily depend on labeled data, overlooking the potential of abundant unlabeled data. Pan-cancer studies often focus on molecular features, neglecting clinical correlations with diagnosis and treatment. Additionally, data imbalance and the underrepresentation of some tumor types lead to unstable models.

Moreover, a lack of standardized benchmarks, limited cross-cohort validation, and a need for uncertainty quantification and calibration remain significant obstacles for the field. The absence of standardized and reproducible benchmarks hampers fair comparison across methods. We encourage the community to establish unified benchmark datasets with consistent splitting protocols—such as 5-fold stratified cross-validation (CV) standardized in TCGA-33 mRNA data with fixed preprocessing steps (e.g., gene filtering, normalization, and batch-effect correction) to facilitate transparent and reproducible evaluation. In addition, the use of common baseline models (e.g., logistic regression, random forest, standard deep neural networks) alongside more advanced architectures will help future studies assess genuine performance gains. Data imbalance, especially the underrepresentation of rare cancers, further restricts the generalizability of the model, calling for strategies such as data augmentation, few-shot learning, or federated learning to mitigate scarcity.

Future studies should prioritize semi-supervised learning (SSL) frameworks to leverage both annotated and unannotated datasets, thereby addressing data scarcity challenges. Self-supervised pre-training on large-scale unlabeled datasets could uncover tumor heterogeneity and enhance downstream classification tasks. Incorporating multi-modal data fusion—combining genomics, proteomics, and normal tissue data—could bridge the gap between molecular research and clinical applications.Beyond general cancer classification, future research must pivot toward more granular, clinically actionable predictions. This includes predicting cancer subtypes, disease stage, patient survival rates, and response to specific treatments, which directly informs personalized medicine.

In conclusion, addressing data limitations, imbalance, and clinical integration using advanced techniques such as SSL and multimodal fusion will enable more robust pan-cancer classification models, improving cancer prediction, diagnosis, and treatment for better patient outcomes.

## 6 Clinical translation and ethics

Developing robust pan-cancer models is the first step; translating them into effective clinical tools requires addressing a second set of critical challenges related to translation, generalizability, and ethics. Although a model may perform well on a single curated dataset, its utility in real-world clinical practice depends on its performance in diverse patient populations and healthcare systems.

Currently Available vs. Necessary Validation. Pancancer models are mainly in the research and development stages. Models that can now be used are typically those integrated into established platforms (like the CGC) for secondary research analysis, offering broad tumor type classification or basic survival predictions on standardized datasets (e.g., TCGA, CPTAC). However, most high-performing models require rigorous, multi-center external validation before they can influence patient care. To ensure external validity, models must be evaluated in data from multiple centers, reducing batch effects and acquisition bias that can arise when trained in the data set of a single institution (Cen et al., 2025). Batch effects, often stemming from variations in sequencing platforms or laboratory protocols across different institutions, can introduce confounding signals that a model may mistakenly learn as biological features. Similarly, acquisition bias can occur if certain rare cancer subtypes or patient demographics are disproportionately represented in the training data from a single center, limiting the model’s ability to generalize to a broader patient cohort.

Equally important is equitable performance across diverse demographic groups. The precision of a model must remain consistent regardless of the race, sex, or age of the patient, to ensure fair clinical outcomes and prevent health disparities from being exacerbated (Desai et al., 2022). These validation efforts must be accompanied by strict attention to data privacy and informed consent, particularly given the reliance of pan-cancer studies on large-scale, sensitive patient data. Concurrently, the increasing complexity of deep learning models highlights a critical need for interpretability, enabling clinicians to understand model predictions and extract meaningful biomarkers that inform clinical decision-making with confidence (Su et al., 2024). Going beyond simply identifying individual genes, interpretable models can provide pathway-level attribution, linking predictions to entire biological processes (e.g., the p53 signaling pathway), which offers more clinically actionable and biologically meaningful insights.

To be reliable for high-stakes clinical decisions, a model must also provide more than a single prediction. It is crucial for models to offer uncertainty estimation, which allows clinicians to gauge the confidence of the model in its prediction. A well-calibrated model, for example, will have its predicted probability (e.g., a 90% chance of a certain tumor type) accurately reflect its true correctness. Such reliability measures are essential to build trust and ensure the safe deployment of these models in patient care. Furthermore, potential regulatory considerations are paramount; any model intended for diagnostic or prognostic use must undergo rigorous review by regulatory bodies (such as the FDA) to ensure safety, efficacy, and clinical benefit.

In conclusion, the path from a pan-cancer model to a clinical tool is complex. It requires a holistic approach that moves beyond technical performance metrics to embrace the crucial factors of external validation, cost-effectiveness, and ethical responsibility. This comprehensive perspective is essential for developing models that are not only accurate in a research setting but are also robust, trustworthy, and beneficial in real-world clinical applications.
