# Correction: Computational models for pan-cancer classification based on multi-omics data

**DOI:** 10.3389/fgene.2025.1743847

**Published:** 2025-12-01

**Authors:** Jianlin Wang, Jiao Zhang, Xuebing Dai, Chaokun Yan, Caili Fang

**Affiliations:** School of Computer and Information Engineering, Henan University, Kaifeng, Henan, China

**Keywords:** pan-cancer classification, multi-omics data, deep learning algorithm, convolutional neural network, tumor heterogeneity

The funder “the Strategic Priority Research Program of Chinese Academy of Sciences (Grant No. XDA0480502)” to Jianlin Wang was erroneously omitted.

The funding statement has been updated as: The author(s) declare that financial support was received for the research and/or publication of this article. This study was supported by the Strategic Priority Research Program of Chinese Academy of Sciences (Grant No. XDA0480502).

The original article has been updated.

